# Using Masao facial makeup in software interface interaction design from the perspective of digital communication

**DOI:** 10.1038/s41598-025-90448-8

**Published:** 2025-03-05

**Authors:** Jinxia Wang

**Affiliations:** Art design College of Shaanxi Fashion Engineering University, Xi’an City, 712046 China

**Keywords:** Masao facial makeup, Interface interaction design, Intangible cultural heritage, Digital communication, User needs analysis, China, Internet technologies, Traditional habits, Folk culture, Questionnaire, Mathematics and computing, Computer science, Information technology

## Abstract

Masao facial makeup, a unique folk cultural form from the Baoji region, carries a rich traditional heritage. However, its inheritance and development have long been hindered by limited dissemination channels and insufficient public attention. This paper proposes an innovative model for deeply integrating Masao facial makeup with computer software, and transforming it into an interactive filter within social media applications. This unique design not only fills the gap in the digital representation of Masao facial makeup but also takes a crucial step in combining folk culture with modern technology. Through a systematic survey, this paper comprehensively collects and precisely analyzes user expectations for functionality and suggestions for improvements regarding the Masao Facial Makeup APP. Based on this, the paper develops a functional system that aligns with user needs. The art essence of Masao facial makeup is then cleverly incorporated into the app’s innovative interface design. A group of 50 representative volunteers are selected to undergo a strict feasibility test of the app, and the APP is then compared to two mainstream traditional cultural apps on the market. The results show that the Masao Facial Makeup APP demonstrates significant advantages. This APP far surpasses the control group in core dimensions such as visual effects, functional attributes, interactive experience, and operational experience, with scores of 4.2, 4.9, 4.5, and 4.6, respectively. During the testing process, volunteers highly praise the app’s interactive interface design and fully recognize the smoothness and convenience of its operation. The key research contribution lies in successfully establishing a new paradigm for the cultural inheritance of Masao facial makeup. By leveraging digital tools, it significantly enhances its dissemination effectiveness and cultural influence, providing an innovative template and practical path for the protection and inheritance of similar folk cultures. This paper strongly promotes the innovative development and sustainable inheritance of folk culture in the digital age and holds the potential to revitalize more cultural treasures with the support of modern technology. It enriches the cultural inheritance ecosystem, and expands the boundaries and possibilities of cultural transmission.

**Supplementary Information:**

The online version contains supplementary material available at 10.1038/s41598-025-90448-8.

## Introduction

Chinese traditional culture is ancient and diverse. However, with the advancement of technology and changes in living environments, many traditional cultures have gradually faded from public view and are even on the brink of extinction. Among them, intangible cultural heritage (ICH) is of particular concern^1^. To effectively protect and inherit ICH, the country has proposed a model combining ICH with internet technology. With the concerted efforts of various parties, significant progress has been made in the protection and inheritance of ICH, and the integration of internet technology has ushered it into a new stage^2^. The widespread use of smartphones has made information access more convenient. Combining ICH dissemination with mobile internet not only reduces communication costs but also broadens the audience, becoming a major direction for future development^3^.

Currently, traditional methods of protecting ICH, such as exhibitions, are struggling to keep up with the rapid pace of technological advancements. The use of mobile devices for the dissemination and promotion of ICH has become a new approach. The app-based ICH promotion and special effects applications have emerged as the main means of promotion on mobile devices^4^. Therefore, to promote this method and contribute to the effective protection of ICH, this paper focuses on the study of ICH special effect dissemination on mobile devices, aiming to advance the development of ICH protection efforts.

In order to better protect and inherit Masao facial makeup, this paper integrates it with internet technology and constructs a digital platform to use a mobile APP for its protection and dissemination. Additionally, based on the unique artistic features of Masao facial makeup, the medium is altered by applying face paint to everyday objects and selling these on the APP, which helps to actively promote its dissemination. Finally, a survey is conducted to assess the current effectiveness of the face makeup APP. The innovation of this paper lies in combining ICH with internet platforms. Without altering the essential artistic characteristics of ICH, the medium is changed to make it closer to people’s daily lives, offering a new research perspective and direction for ICH inheritance. This approach is expected to provide valuable insights for the protection and inheritance of other ICH projects, helping traditional culture thrive in modern society and achieving an organic unity of sustainable development and innovative inheritance.

## Related works

In recent years, the international community and China have paid more and more attention to the protection and inheritance of ICH, and there are more and more corresponding studies. Su et al. reported that, with the extensive attention of scholars on ICH, research on ICH had greatly increased. Their research focus was mainly on creative intangible tourism, community participation in intangible protection and development, and studies on the authenticity of ICH^5^. Koutsabasis et al. introduced the addition of ICH elements to location-based games. This approach aimed to promote the understanding and learning of ICH, particularly crafts and handicrafts, throughout the entire long-term project, from publicity to implementation^6^. Hou et al. designed and implemented a digital online display system for ICH based on mobile augmented reality technology, aiming to address the problems of limited formats and poor interactivity in ICH protection. They designed the main functional modules for the system’s cloud and mobile terminals. A rapid modeling method was proposed, combining 3D scanning with iterative mesh simplification. This method improved the fidelity and smoothness of ICH products displayed on mobile terminals. Additionally, it enabled real-time interactive coloring and display after the model was enhanced, which significantly increased the fun and interactivity of ICH protection and dissemination^7^. Li and Duan discussed the protection model of ICH by integrating the concept of “Internet Plus.” This approach provided practical guidance for establishing a new “Internet Plus ICH Protection” model and introduced innovative ideas and methods for ICH protection^8^. With increasing national attention to ICH, China has developed its own unique working model for ICH protection based on the foundational theoretical framework provided by UNESCO^9^.

In summary, Masao facial makeup, as a folk culture popular only in certain regions, has long been overlooked by most orthodox scholars. This phenomenon has directly led to a severe lack of information related to the study and documentation of the Masao facial makeup. The few existing studies on Masao facial makeup are mostly limited to exploring its historical origins and development, while research on its artistic and cultural values remains very weak, almost in its infancy. A further critical analysis reveals that this research status has many drawbacks. First, the lack of in-depth exploration of its artistic value has prevented the Masao facial makeup from being fully recognized for its unique position in the field of art. Its distinctive design, use of color, and symbolic meanings should occupy an important place in the study of folk art. However, due to insufficient research, these valuable artistic resources have not been effectively referenced or applied in broader artistic creation and theoretical research. Besides, regarding cultural value, the rich cultural connotations embodied in the Masao facial makeup, such as local folk beliefs, social customs, and traditional values, have not been systematically sorted out or explained. This not only hinders the comprehensive understanding of regional cultural diversity but also leaves the Masao facial makeup without solid theoretical support in the process of cultural transmission and dissemination. Moreover, this makes it difficult to achieve effective protection and development in the contemporary cultural context. Future research should aim to reverse this situation. It should broaden the research perspective, and apply multidisciplinary methods, such as art studies, cultural anthropology, and folklore, to comprehensively and deeply analyze the artistic and cultural values of the Masao facial makeup. This can fill the gaps in current research and lay a solid theoretical foundation for the inheritance and development of Masao facial makeup culture.

## Methodology

### Overview of Masao facial makeup

Shehuo is a traditional carnival activity of Chinese Han people to celebrate the Spring Festival. In Shehuo activities, traditional masks are used as performance props. Masao facial makeup, which originates from the Shaanxi folk fire mask, gradually evolved into a distinct local folk handicraft. Known for its vibrant colors, it is highly decorative. The composition of the makeup is highly flexible, with multiple spectral patterns that can be combined and altered to create diverse structures. The main patterns on the forehead, nose, and center of the mask are designed by local artisans based on their own ideas. Each pattern carries a unique symbolic meaning, collectively reflecting people’s deep yearning for a better life.

**Fig. 1:**
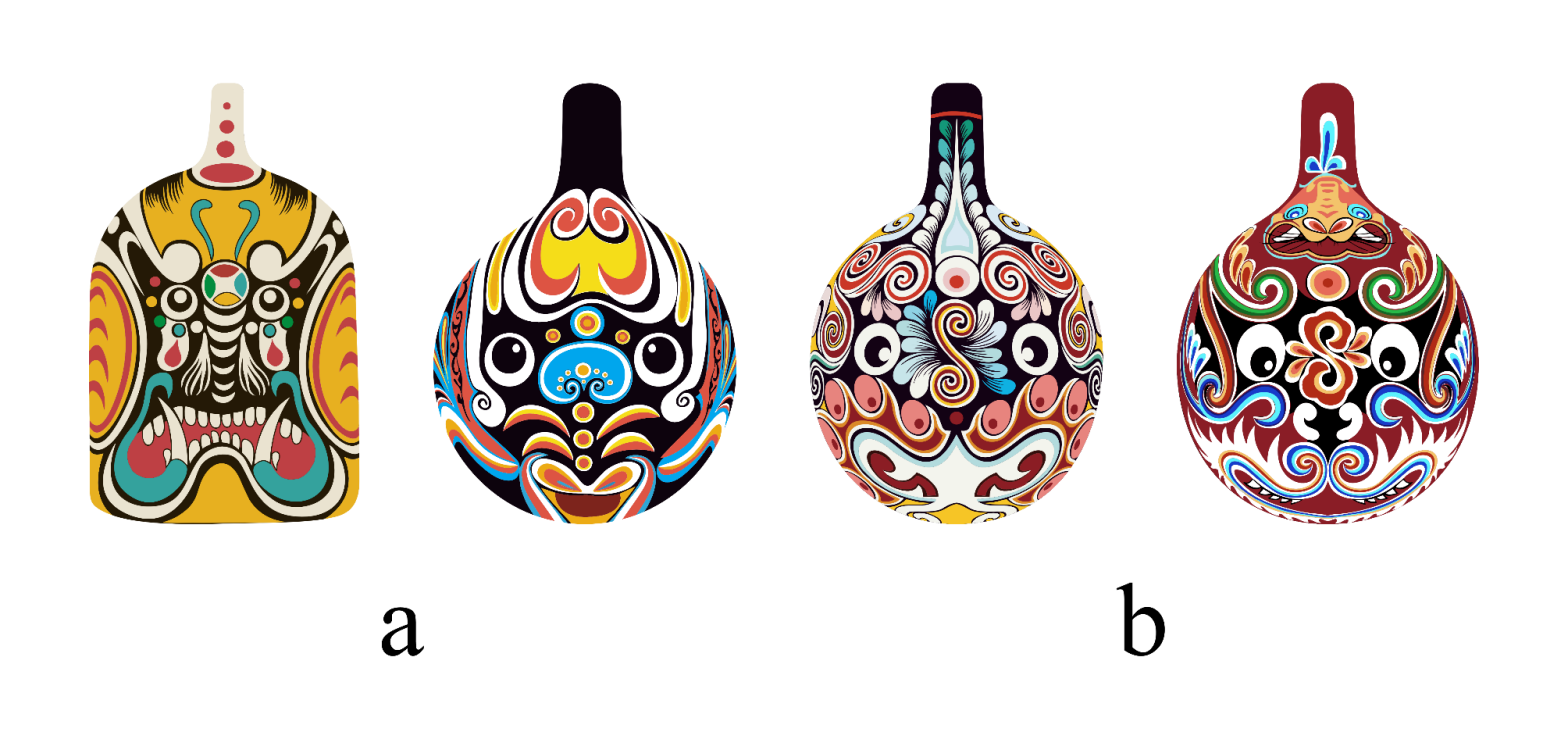
Masao facial makeup character facial image (**a**) Symmetrical type (**b**) Rotational type.

The common face shapes in the Masao facial makeup are the symmetrical type and the rotational type, with the symmetrical type being the most prevalent. Typically, most Masao facial makeup presents upright character images. To illustrate this more clearly, Fig. 1 shows clear examples of the symmetrical and rotational types of this makeup, which helps readers visually understand these characteristics.

**Fig. 2:**
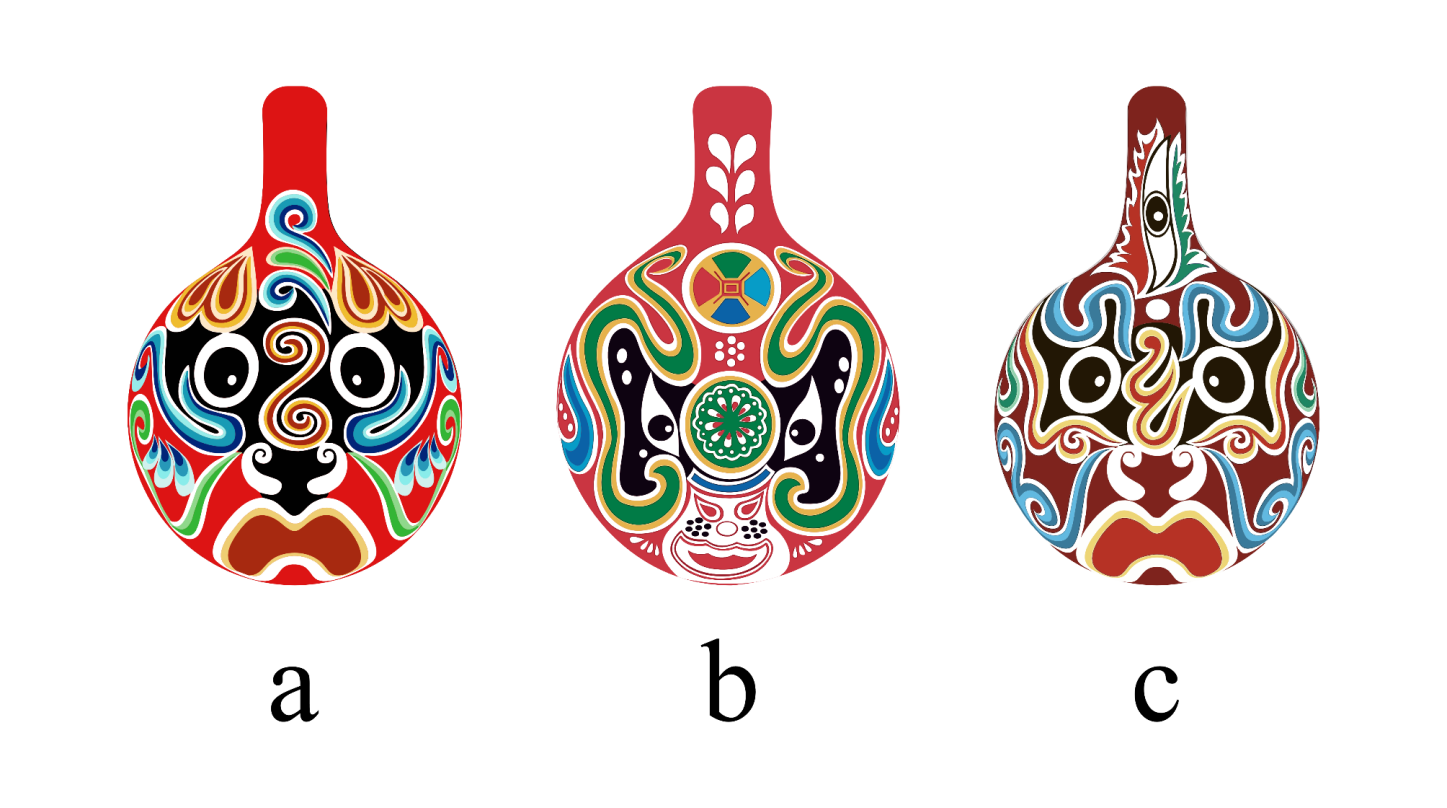
Masao facial makeup’s eye shape (**a**) leopard eye (**b**) water hyacinth (**c**) smart eye.

Figure 1 shows the overall appearance of the Masao facial makeup, with the most striking feature being the exaggerated design of the eyes. This unique design is full of artistic charm. To help the audience better appreciate the distinctive features of the eye design, Fig. 2 provides a detailed display of the different forms of eyes in the Masao facial makeup, including representative styles such as leopard eyes and phoenix eyes. This allows the audience to deeply experience the artistic appeal of these designs.

**Fig. 3:**
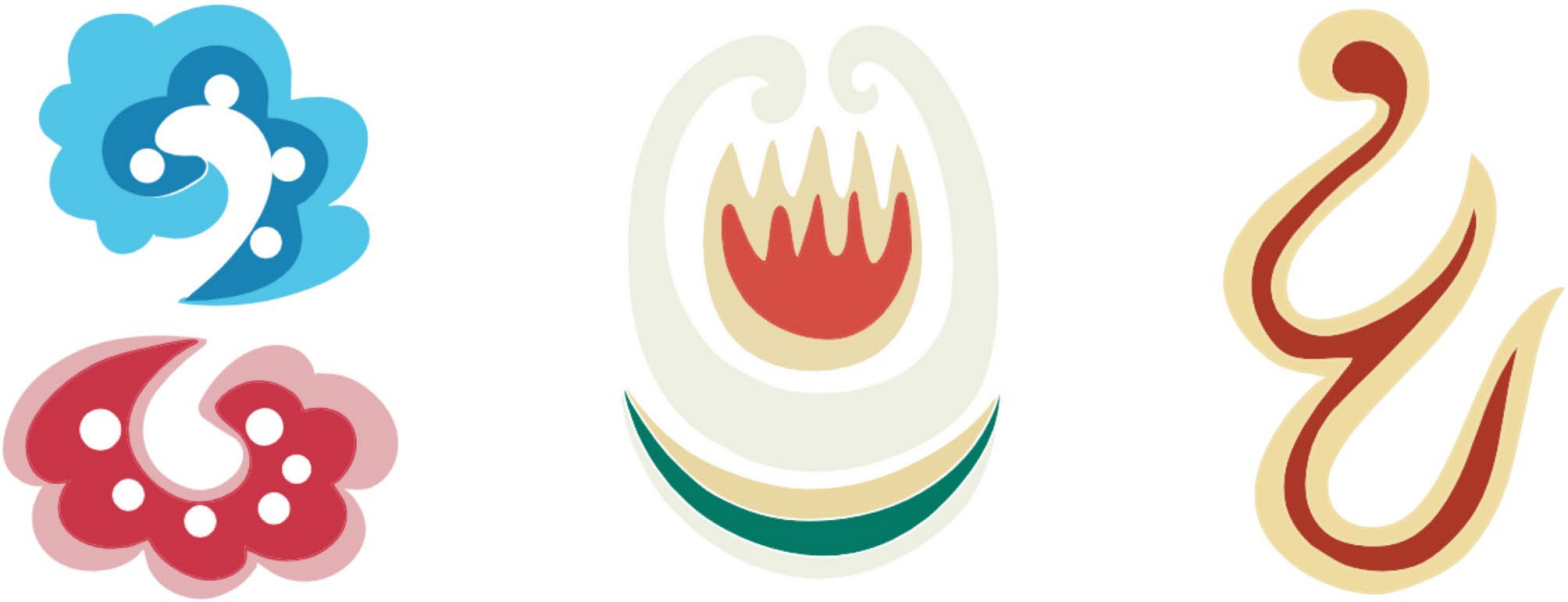
Masao facial makeup of nose flowers with auspicious lines.

Figure 2 shows the special auspicious pattern on the nose area of Masao facial makeup, commonly referred to as the “nose flower”. The presentation of the nose flower plays a key role in the overall facial effect of this facial makeup. Its form can be subdivided into categories such as cloud patterns, Taiji patterns, and comb patterns. To further clarify the specific characteristics of these nose flower designs, Fig. 3 clearly presents the various types of nose flower patterns. This aids in the in-depth study of the composition and artistic features of the Masao facial makeup.

In Fig. 3, as ICH is constantly mentioned, more people are paying attention. There are more and more derivatives related to ICH, and they gradually enter people’s lives and are welcomed by the general public^10^. Combining traditional culture with practical products is closer to people’s lives. The carriers of Masao facial makeup derivatives are mainly daily necessities such as tea sets, ceramics, mirrors, and pendants, which people deeply love.

### Demand analysis and functional positioning of Masao facial makeup APP

**Fig. 4:**
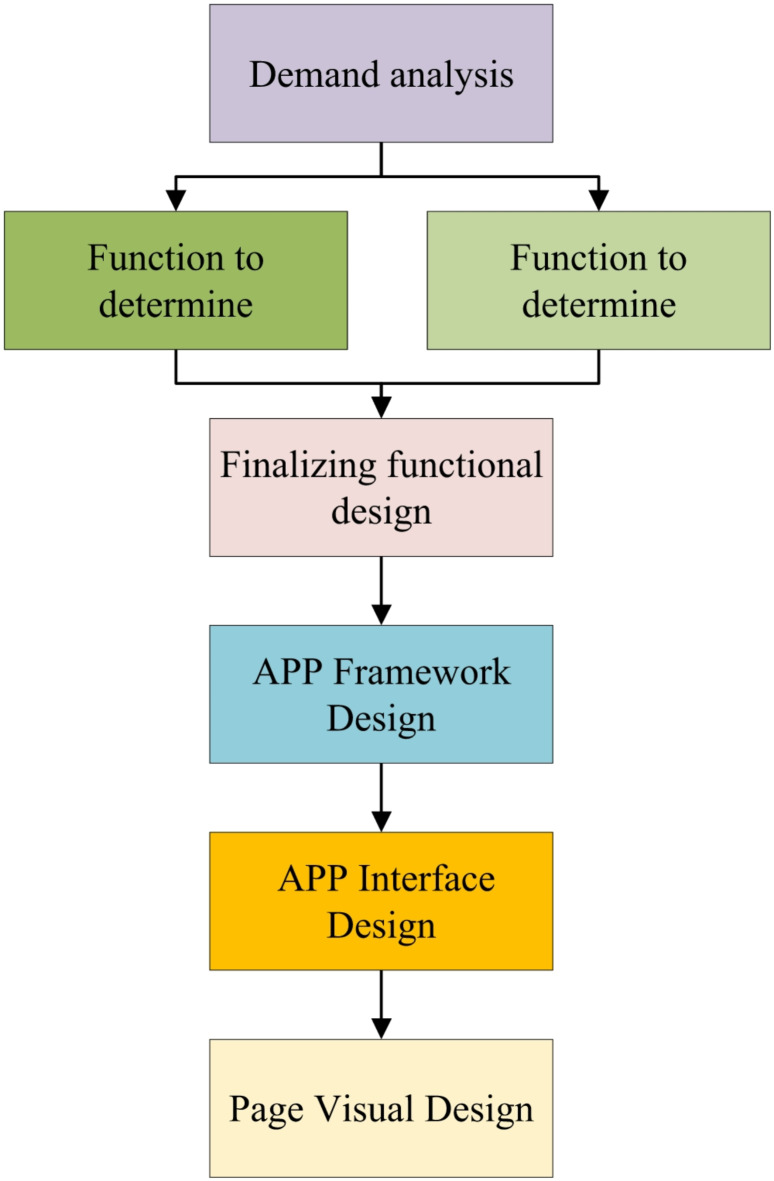
Development process of Masao Facial Makeup APP.

The core of APP development lies in user experience, which essentially refers to the perceptions and responses generated by users during their interaction with the product^11^. When constructing an APP that holds practical value and vitality, it is crucial to prioritize user behavior as the core focus of research based on the fundamental guidelines of interface interaction design^12^. The development process begins with an in-depth analysis of user needs, which are then precisely translated into corresponding functional elements^13^. These functionalities are subsequently systematically organized and categorized, forming a scientifically rigorous and logically clear framework. Based on this framework, the page flow is generated through its inherent page logic, gradually outlining the prototype of the APP. Finally, detailed page design is carried out based on the carefully crafted prototype, transforming the product from blueprint to reality, thus completing the final APP development^14^. Figure 4 illustrates the complete development process.

Figure 4 shows the key role played by Adobe XD in the design process of the Masao Facial Makeup APP. It offers powerful interface design capabilities, allowing designers to efficiently create static page layouts for the APP by utilizing its extensive component library and intuitive interface. Designers can precisely define the position, size, color, and style of elements, ensuring consistency and aesthetic appeal in the interface design. This results in a clear and comfortable visual experience for users, meeting their visual experience requirements. ProtoPie, on the other hand, focuses on creating interactive prototypes. With its visual programming environment, designers can easily create various interactive behaviors such as page transitions, element animations, and gesture operations without complex coding. This enables designers to quickly validate the APP’s interaction flow and functional logic in the early development stages, identify issues, and make improvements in a timely manner. This ensures the smoothness and ease of use of the final application in terms of both functionality and interaction, enhancing the user experience and better realizing the APP’s various functions. It also facilitates the effective dissemination and inheritance of Masao facial makeup culture on digital platforms.

### Analysis of needs

ICH represents the invaluable legacy of each era and serves as a historical resource that connects the past with the present. The protection and dissemination of ICH cannot rely solely on traditional media. It is only through the integration of advanced science, network information, and new media technologies that ICH can be more widely understood. By leveraging the network information technologies embedded in computers and mobile devices, ICH can be effectively preserved, protected, and shared with a broader audience in a realistic and accessible manner^15^.

The protection of ICH has become increasingly urgent. Preserving ICH requires dedicated inheritors, but the rapid advancement of science and technology has left many forms of ICH without successors. Most existing inheritors are aging, which has led to the gradual fading of certain ICH traditions from daily life^16^. To ensure the continued transmission and wider dissemination of Masao facial makeup, an ICH App will showcase this unique cultural heritage to both traditional culture enthusiasts and lovers of Masao facial makeup through new media platforms. This approach will help more people understand and actively engage with this facial makeup culture^17^. The development of the ICH APP begins with the strategic layer of its architecture. A thorough and detailed needs analysis is conducted to clarify the purpose of the APP. Additionally, user surveys and feedback help shape the APP’s infrastructure and identify the appropriate functional modules^18^.

1. Product positioning
  A decisive factor determining the success or failure of an APP is the clear positioning of the product itself. The principle of sustainable development in ICH protection requires that the APP’s interface design be fully user-centered, ensuring that the product is always developed with the user’s needs in mind. By understanding both user needs and product goals, the design can effectively meet the requirements of both^19^. The product positioning of this design is an all-encompassing application for showcasing ICH information, centered around Masao facial makeup. The goal of the APP is to provide a multimedia platform for the dissemination and learning of Masao facial makeup. It intends to offer users a comprehensive resource for exploring folk culture and traditional craftsmanship associated with this facial makeup^20^.
2. Questionnaire survey.
  The purpose of the survey is to use a questionnaire to investigate users’ needs. The analysis of the questionnaire data determines the user’s needs and provides the infrastructure for determining the functions of the Masao Facial Makeup APP. Table 1 displays the content of the questionnaire.

**Table 1:** Contents of the demand questionnaire.

Question	Answer
1. What is your gender?	(A) male (B) female
2. What is your age?	(A) Under 20 years old (B) 21–35 years old (C) 36–45 years old (D) Over 46 years old
3. What is your occupation?	(A) staff of government enterprises (B) private enterprises (C) foreign enterprises (D) other
4. What system mobile phone do you use?	(A) Apple system (B) Android system (C) Other
5. Do you know about Masao facial makeup?	(A) don’t know (B) a little (C) know (D) know a lot
6. What is the biggest problem facing Masao facial makeup protection?	(A) Lack of perfect protection mechanism (B) Insufficient dissemination (C) Lack of inheritors (D) Lack of funds
7. Would you like to download the Masao Facial Makeup APP on your mobile phone?	(A) willing (B) not willing
8. How did you come into contact with Masao facial makeup?	(A) Reading and newspapers (B) Watching TV (C) Internet (D) School study (E) Travel (F) Others
9. What do you value more about Masao facial makeup?	(A) Interesting (B) Educational significance
10. What is the most important element of using the APP?	(A) Rich in content (B) Simple and smooth operation (C) Complete functions (D) Strong interaction (E) Beautiful interface (F) Others
11. If there is a Masao facial makeup protection APP, what functions do you want it to have?	(A) Short video (B) Manual tutorial (C) Offline experience course (D) Community communication (E) ICH product sales function
12. Have you used apps from other traditional cultures?	(A) Yes (B) No
13. What are the shortcomings of the existing traditional cultural APPs?	(A) The content is not rich enough. (B) The interface is not beautiful (C) The lack of attractiveness (D) False propaganda (E) The interaction is poor (F) The function is relatively backward

**Fig. 5:**
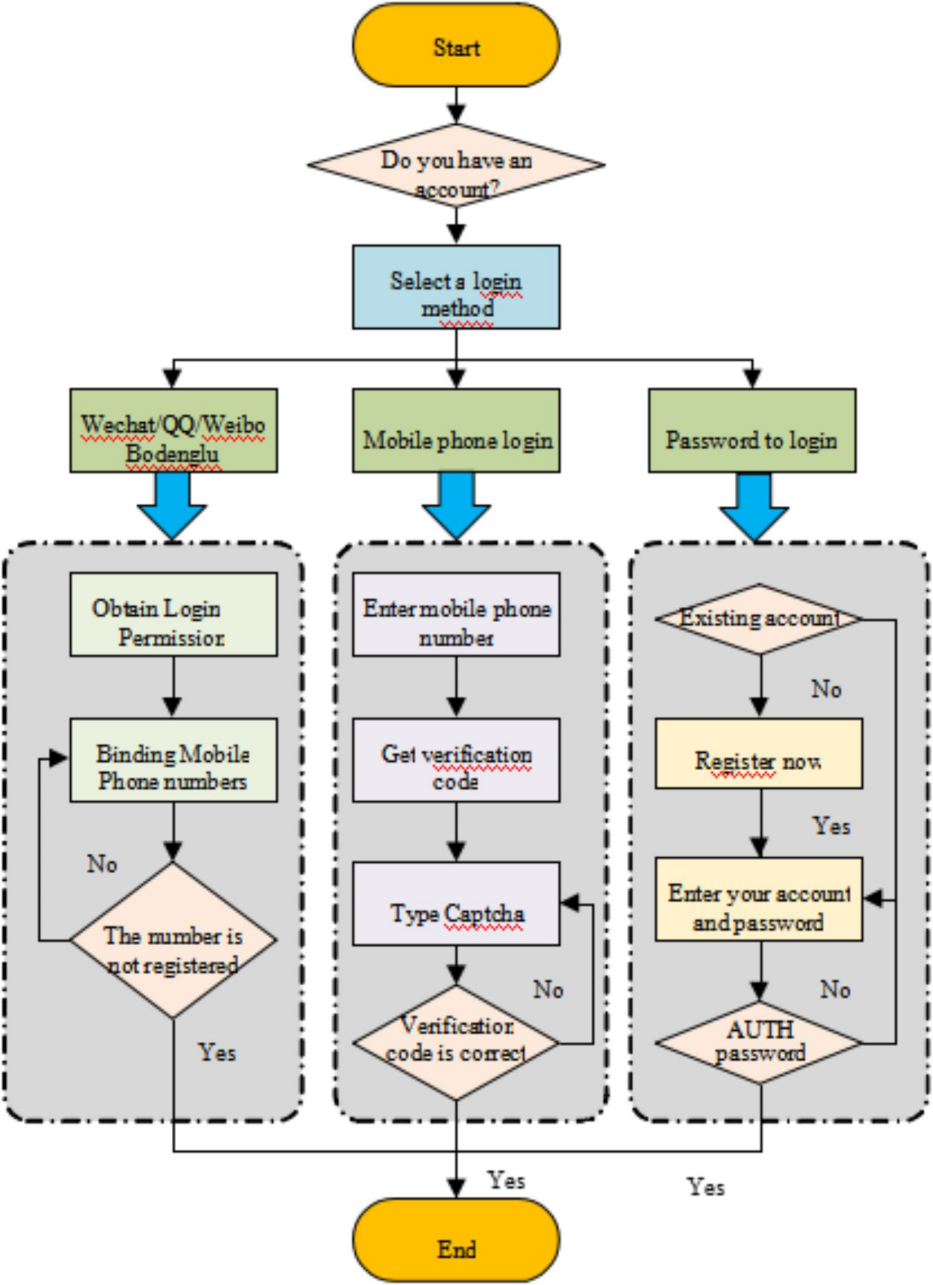
The APP login process.

The paper employs a specially designed questionnaire, covering multiple dimensions such as visual effects, functional usability, interactive experience, and content richness. Each dimension is further divided into specific questions. For example, the visual effects section includes questions on color matching and pattern clarity, while functional usability addresses issues such as the completeness of features and ease of operation. These detailed questions are used to comprehensively collect volunteers’ feedback and opinions on their user experience. In the data analysis, the responses are first organized and coded. After removing invalid and incomplete questionnaires, statistical methods are applied to calculate descriptive statistics such as mean and standard deviation for each indicator. It aims to provide an overview of volunteers’ overall evaluations of the Masao Facial Makeup APP and the comparison APP across various dimensions, and the degree of variation. Correlation analysis is also used to explore potential relationships between different dimensions, such as the connection between visual effects and user satisfaction. Finally, the complex analysis results are presented in a simplified and intuitive manner in Fig. 5. The charts clearly highlight the advantages and disadvantages of the Masao Facial Makeup APP compared to the other two APPs in each evaluation metric, offering strong data support and directional guidance for further optimization of the APP. This ultimately contributes to better preserving and promoting this facial makeup culture on digital platforms.

This paper uses a multi-stage stratified random sampling method to select a representative sample of volunteers. First, the research area is divided into several sub-regions based on geographical distribution, including urban core areas, urban-rural fringe zones, and surrounding towns. Within each sub-region, stratification is further carried out according to the type of venue. Sampling points are chosen in locations with significant foot traffic and varied cultural atmospheres, such as local cultural centers, schools (from elementary to university), community activity centers, and traditional markets. During the recruitment process, the research team sets up fixed recruitment booths at each sampling point and assigns trained surveyors. The surveyors provide potential volunteers with detailed explanations of the research background, objectives, and the importance of completing the questionnaire for the inheritance and development of Masao facial makeup culture. To ensure volunteers fully understand the participation requirements, they also explain the following aspects: (1) the estimated time for completing the questionnaire (approximately 15–20 min); (2) the general scope of the questionnaire (covering knowledge of Masao facial makeup, interest level, exposure methods, and expectations for related APP functions); (3) the voluntary nature and confidentiality principles of the study. For volunteers who agree to participate, the surveyors assign a unique identification code using a random number generator. Based on pre-established random allocation rules, the volunteers are assigned to either the experimental or control group, ensuring both groups are balanced and comparable in terms of basic characteristics. After a period of recruitment, 282 completed questionnaires are collected. Two independent data entry personnel input the questionnaire data into a specially designed database. Obvious logical errors (such as unreasonable ages or responses where all questions are answered with the same option) are flagged during the data entry process. After the data entry is completed, an automated data cleaning program, combined with manual verification, is used to rigorously check the completeness, logical consistency, and internal consistency of the questionnaires. After eliminating invalid responses, 276 valid questionnaires are identified as the basis for further data analysis. Before the study begins, each volunteer receives a detailed informed consent form that outlines the specific research process, potential risks and benefits, privacy protection measures, and the right to withdraw from the study at any time. After reading and understanding the consent form, volunteers sign it, confirming their agreement to participate. This ensures that the study follows ethical standards and legal requirements. All aspects of the study, from questionnaire design, distribution, and collection, to data entry and analysis, adhere to standard academic research protocols, ensuring the scientific rigor, reliability, and validity of the research. This provides a solid data foundation and methodological guarantee for further in-depth studies on Masao facial makeup.

### Positioning of Masao facial makeup

**Fig. 6:**
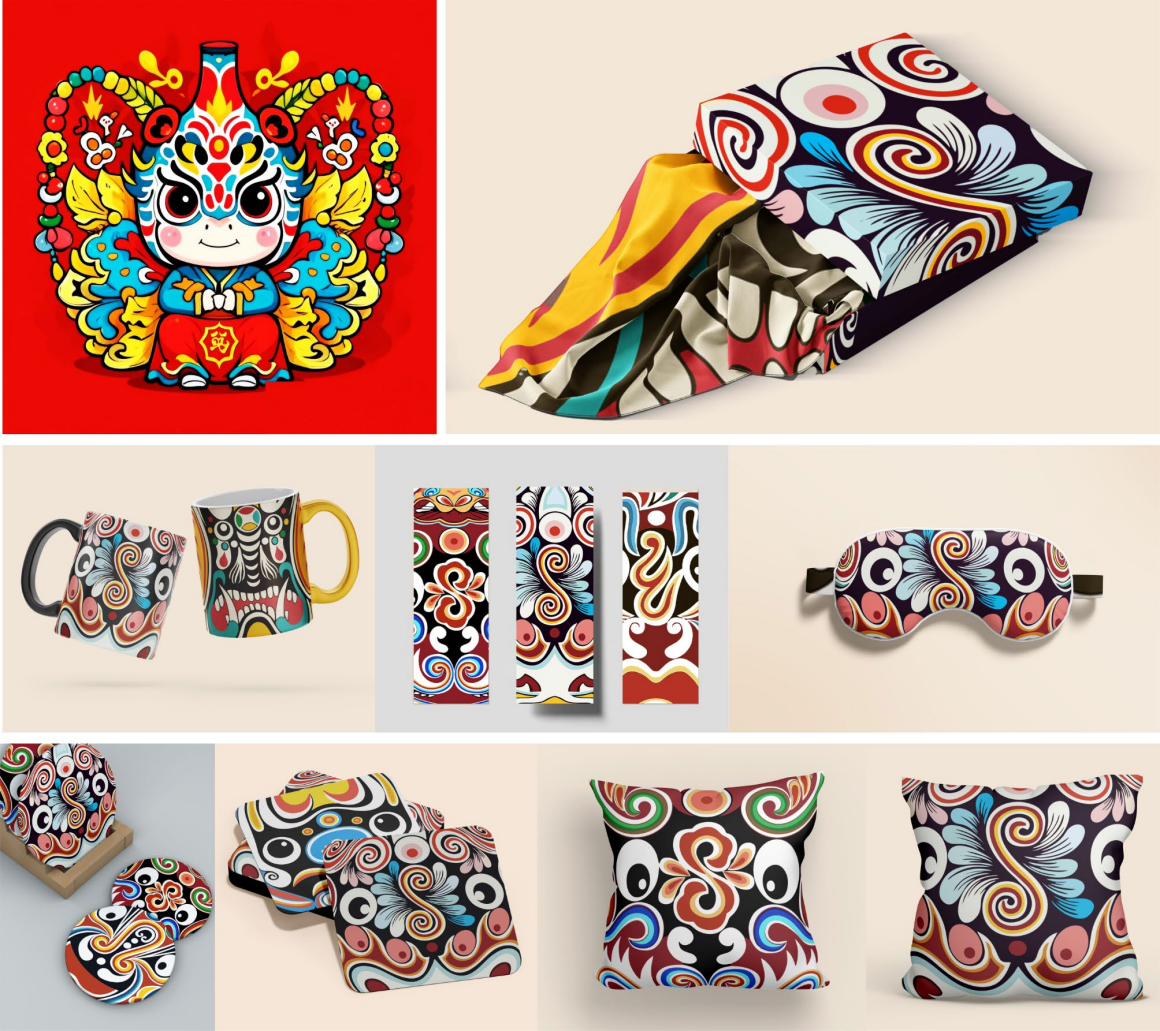
Derivatives of Masao facial makeup.

The target user group for the Masao Facial Makeup APP is people aged 15 to 45, particularly those who have a passion for traditional culture and are interested in cultural inheritance. In the current age of information overload, the internet has become a crucial channel for people to acquire new knowledge. The Masao Facial Makeup APP serves as such a platform providing high-quality information to users. According to the results of a volunteer survey, many users, while learning about local folk culture, also have a need to explore travel guides. The APP meets this need by offering a wealth of related information. Specifically, users can watch short videos about Masao facial makeup on the platform and share their travel experiences and insights by posting videos or commenting. Furthermore, users can purchase Masao facial makeup-related products within the APP or use its location features to visit offline physical stores. To provide a more intuitive view of the derivative products of Masao facial makeup, Fig. 6 showcases various forms of its derivatives, including crafts designed with such elements. This not only enhances understanding of the application of Masao facial makeup in the cultural industry but also highlights its potential for further expansion.

APP function positioning. The functions are mainly reflected in the Masao facial makeup’s information display, short video function, community communication, commodity purchase, and other functions. Users can understand the related customs and culture by going to the corresponding location based on the positioning map. The information function of the proposed APP understands the latest information and learns the core knowledge based on online classes. The online sales platform sells Masao facial makeup-related products.

### App usability testing

To assess the usability of the Masao Facial Makeup APP, a test is conducted with volunteers who interact with the app’s interface. A total of 50 volunteers, aged between 15 and 45, are invited to participate in the test. The participants come from diverse occupational backgrounds, including students, designers, and professionals involved in traditional culture. Additionally, two other traditional culture-related Apps are included for comparison. The evaluation is carried out through a questionnaire, focusing on three main aspects: visual presentation, functional attributes, and interface interaction experience. The volunteers rate each aspect using a Likert scale, with scores ranging from 1 to 5, corresponding to very poor, poor, average, good, and very good, respectively. Before the evaluation, an introduction to the APP’s main functions is provided, and volunteers’ ratings are collected after their evaluation.

## Results and discussion

### Questionnaire data analysis results

In the experimental design, the control group consists of individuals who meet specific criteria. Specifically, the selection of these individuals is based on their cultural background and mobile application usage habits, which resemble those of the target audience. Importantly, they have no prior exposure to the Masao facial makeup-related content. This group serves as a comparison group in the study, providing a crucial reference point for accurately assessing the effectiveness of the Masao Facial Makeup APP. The experimental group, on the other hand, is determined through a rigorous selection process. During the extensive volunteer recruitment phase, many potential participants with an interest in traditional culture are attracted. After further screening, the final participants for the experimental group are selected. From an age perspective, 53% of the volunteers involved in the survey are under the age of 20, and 39% are between the ages of 21 and 35, with the majority of participants being young people. In terms of occupation distribution, students represent the largest proportion, followed by young professionals. This age and occupation distribution pattern provides important insights into the acceptance, usage patterns, and potential impacts of the Masao Facial Makeup APP among the younger generation.

To gain deeper insights into the volunteers’ attitudes and expectations towards the Masao Facial Makeup APP, survey data collected from the questionnaire reveals that 82% of participants express a clear intention to download the APP. This high percentage strongly supports the feasibility of advancing this research from the perspective of market demand. Further analysis of the volunteers’ functional expectations for the APP shows that they generally believe that an APP capable of meeting their needs should focus on ensuring content richness. It should include comprehensive and in-depth knowledge of Masao facial makeup culture, diverse presentation formats, and abundant interactive materials. In terms of operational flow, the APP should be simple and smooth to use, minimizing the complexity and waiting time for users. It should ensure a natural and efficient transition between functions, while the functional modules should be complete, covering key areas such as cultural display, learning guidance, and social interaction. Particularly, volunteers emphasize the need for strong interactive features to facilitate communication and sharing among users, and deep interaction between users and the APP’s content. The APP should also feature an attractive interface design that enhances the user experience from a visual standpoint. Among the core content elements, Masao facial makeup short videos and detailed craft tutorials are highlighted. These videos should present every step in the creation of Masao facial makeup, from material preparation and painting techniques to finishing touches. Besides, they need to explain the cultural connotations embedded in its unique artistic style. For example, the cultural meanings represented by color combinations and the historical heritage reflected in the pattern designs should be conveyed.

Furthermore, the survey also reveals the current state of the traditional culture APP market: 52% of the volunteers have never downloaded any traditional culture-related APPs before. When providing feedback on existing traditional culture APPs, most respondents point out issues such as shallow content, a lack of variety in presentation formats, limited depth of exploration, poor interface design, and suboptimal user experience. Based on the survey results and research background, this paper has carefully established both a control group and an experimental group, and has systematically carried out a series of in-depth research activities. Specifically, during the APP usage process, detailed records are kept of the actions of both groups, including but not limited to the frequency of function usage, usage duration, and selection of operational paths. Comprehensive feedback on their user experience is collected, covering subjective evaluations of the interface design, content quality, and interactive features. Additionally, the survey tracks their learning outcomes after using the APP, such as improvements in their knowledge of Masao facial makeup culture and the acquisition of related production skills. Through the detailed analysis of this rich dataset, the goal is to precisely assess the actual effectiveness and mechanisms of the Masao Facial Makeup APP in meeting users’ diverse needs and efficiently passing on the essence of Masao facial makeup culture. This can provide a solid data foundation and guidance for subsequent optimization strategies, continuously improving the quality of user experience and expanding the scope and depth of cultural dissemination. Ultimately, this can contribute to the innovative development and widespread popularization of Masao facial makeup as a unique traditional cultural form in the digital era.

**Fig. 7:**
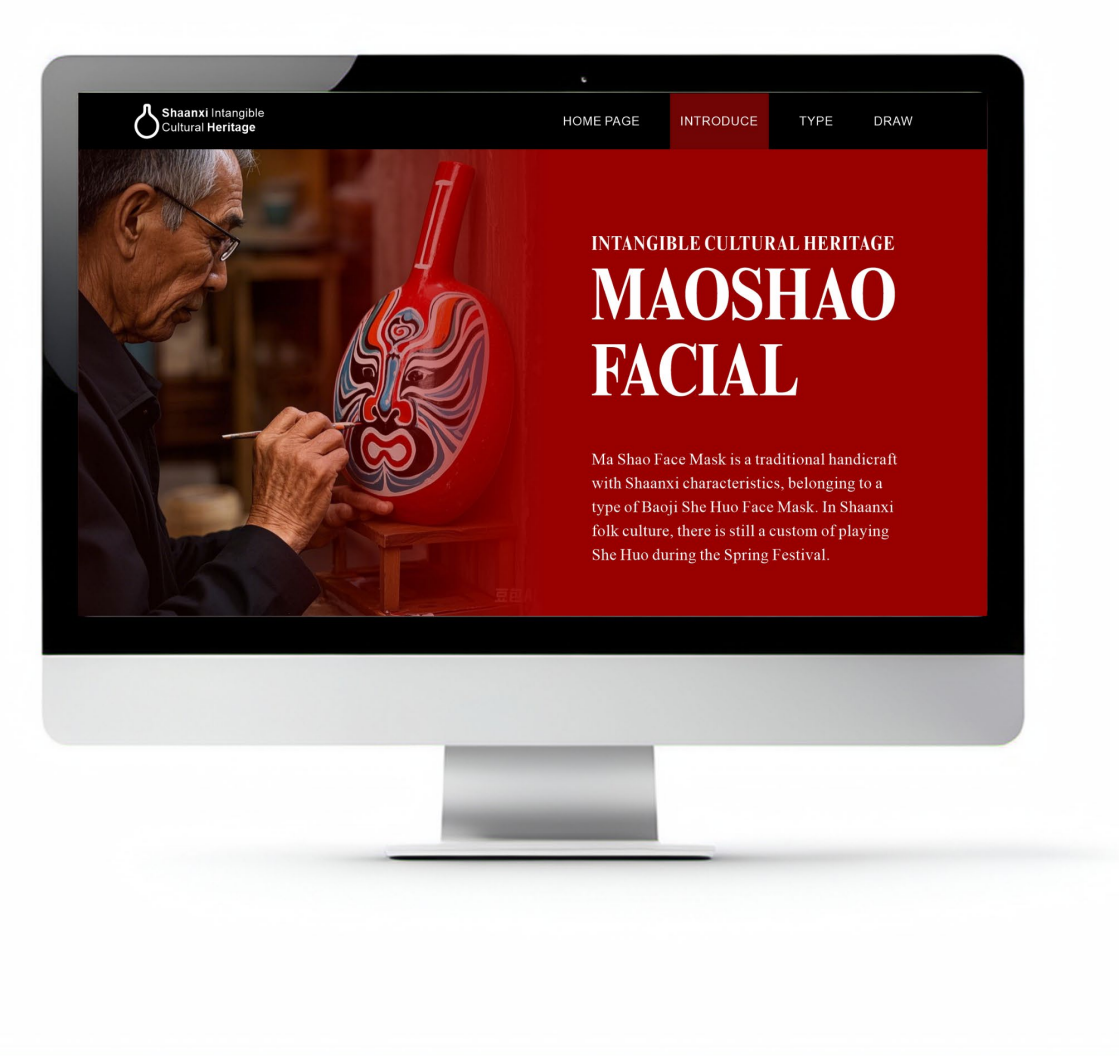
APP interface.

Based on the analysis of user needs, Fig. 7; Table 2 present the statistical results of the APP’s interface and functionalities.

**Table 2:** Determination of user needs and functions of Masao Facial Makeup APP.

Function number	Description of requirement	Exclusive function
1	Watch and upload short videos	Masao facial makeup short video
2	Learn and understand Masao facial makeup	Masao facial makeup online class
3	Users can share videos and pictures	community exchange
4	Users can purchase Masao facial makeup derivatives	Masao facial makeup products for sale
5	The user maintains and manages personal information	Personal information maintenance
6	Can view other people’s comments and user’s comments	Comments and news notifications
7	Users can learn about Masao makeup facial knowledge through short videos	The heir’s documentary video

###  Interaction design of Masao facial makeup APP interface

**Fig. 8:**
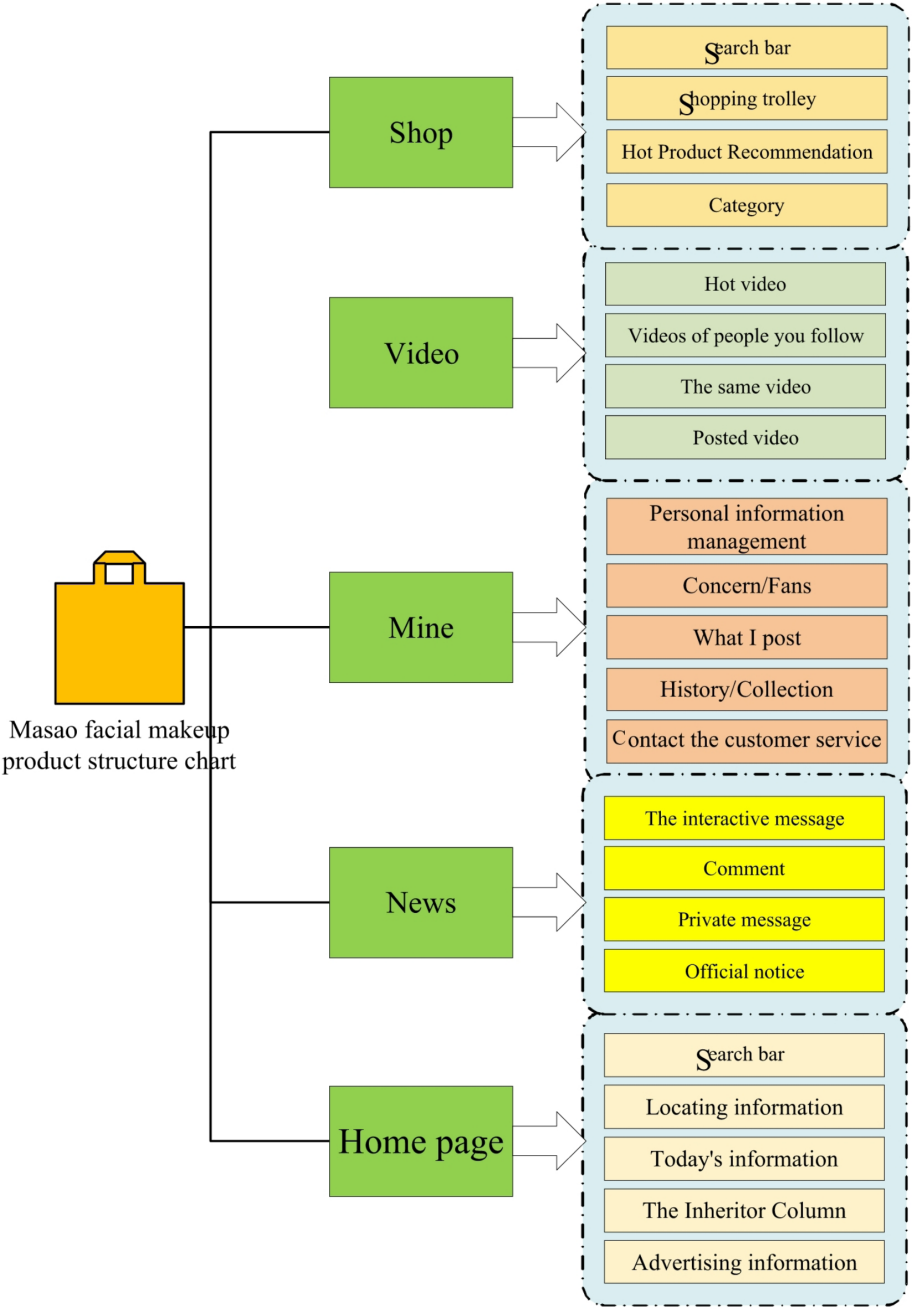
Product structure of Masao Facial Makeup APP.

By thoroughly analyzing user needs and the basic requirements of the product, the fundamental framework of the product is established. Based on this framework, this paper designs a mobile application aimed at preserving and inheriting the Masao facial makeup culture. To best meet public demand, the design process focuses on fulfilling user expectations. The interface is designed for the Android system, following a minimalist style, and professional design software such as Photoshop, ProtoPie, Adobe XD, and Adobe Illustrator is used. Figure 8 presents the product structure of the Masao Facial Makeup APP. This figure clearly shows the layout of the various functional modules, the information architecture, and the relationships between the different parts. It helps to deepen the understanding of the design concept and implementation approach, providing an intuitive reference for subsequent research and optimization.

**Fig. 9:**
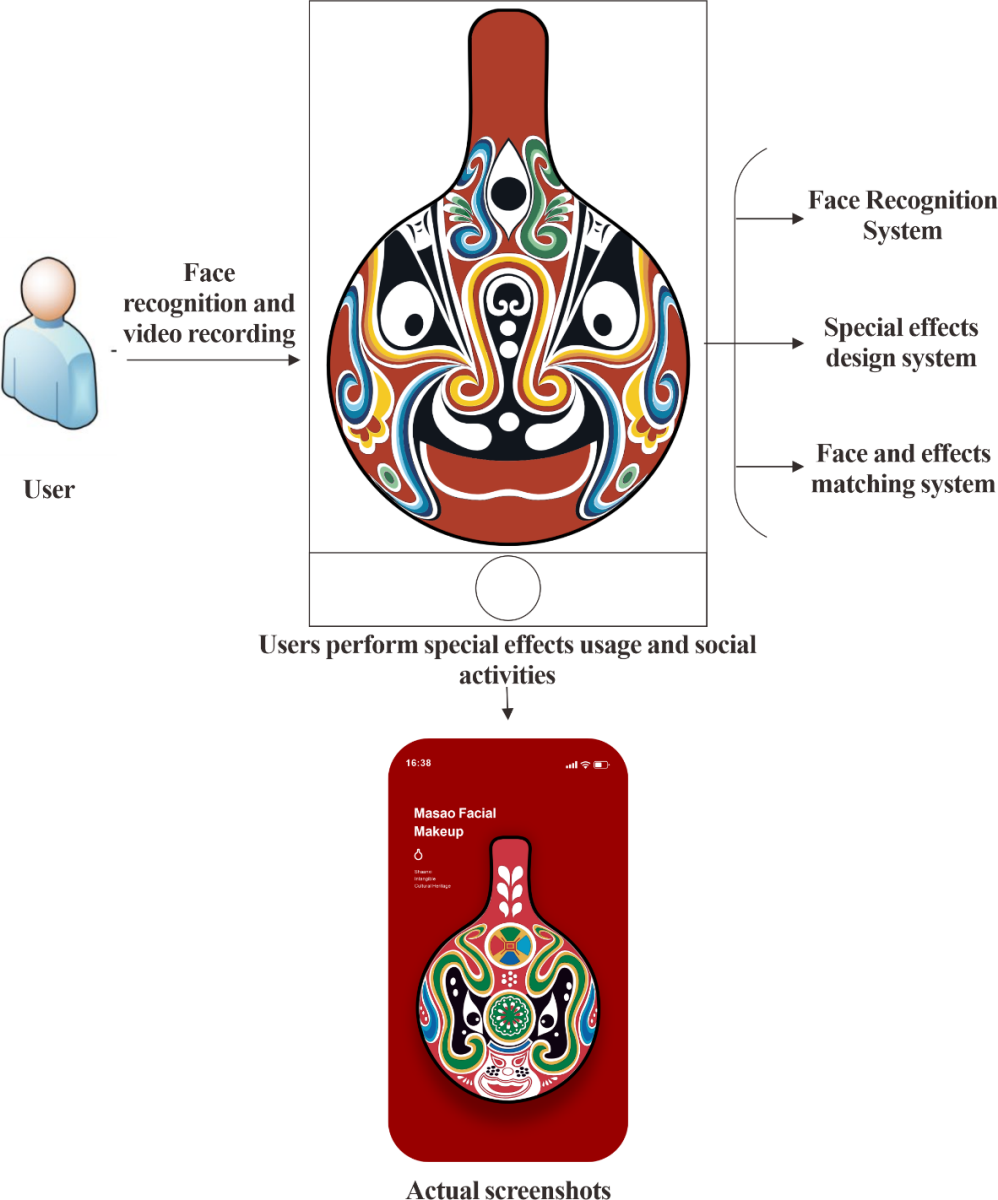
APP structural design.

In Fig. 8, after an in-depth analysis of the basic stable framework and structural content of the APP based on the above discussions and design, this paper demonstrates the feasibility of applying Masao Facial Makeup APP in social software. Meanwhile, the design work of the APP based on this framework and structural content has greatly advanced the technological aspects of this research. Figure 9 is created to clearly present the basic structure of the APP designed. It details the architectural relationships, hierarchical settings, and distribution of functional modules within the APP. This helps further explore the internal operation mechanism and design logic of the APP, providing an important visual reference for subsequent research and analysis.

In Fig. 9, the system primarily consists of three components: the face recognition system, the special effects design system, and the face-special effects matching system. These three systems collectively facilitate the comprehensive design of Masao facial makeup special effects within the APP. Users can then engage with these special effects to participate in social activities through the APP.

In the development of the Masao Facial Makeup APP, the integration of facial recognition technology is one of the key components. By leveraging advanced image recognition algorithms, the APP can accurately identify key facial landmarks through the extraction and analysis of user facial features. This enables the precise alignment of Masao facial makeup effects on the user’s face. Achieving this requires not only efficient feature extraction algorithms but also complex image deformation and mapping techniques, ensuring that the facial patterns synchronize seamlessly with the user’s facial movements, thereby providing an immersive experience. The design of dynamic transition effects further enhances the interactivity of the APP. Using a smooth animation framework, dynamic effects such as fading, scaling, and translation are applied during page transitions and element displays, enhancing visual guidance and making the user experience more fluid and comfortable. For instance, when users navigate from the homepage to the Masao facial makeup display page, a fade-in/fade-out transition gently presents the content, avoiding any abrupt visual changes. Additionally, the optimization of code structure and graphics rendering pipeline reduces lag and flickering, ensuring stable performance across different mobile devices. This guarantees a high-quality interactive experience for users, perfectly blending technology with cultural content and supporting the digital preservation and promotion of Masao facial makeup culture.

In the statistical analysis of usability test scores, the first step is to organize the collected test scores for the Masao Facial Makeup APP. For dimensions such as visual effects, functional attributes, interaction experience, and usability, the mean, median, and standard deviation are calculated. The mean reflects the average evaluation level of users for each dimension, while the median represents the middle value, helping to mitigate the impact of outliers. The standard deviation shows the degree of dispersion, providing insight into the consistency or divergence of user evaluations. Next, correlation analysis is conducted to explore the relationships between dimensions. For example, if a significant positive correlation is found between visual effects and interaction experience, it may indicate that the two are interrelated, with superior visual presentation enhancing the interaction experience. Hypothesis testing is then applied to determine whether there is a significant difference between the scores of this APP and traditional APPs, validating its advantages. Finally, the results of these statistical methods are integrated to rigorously and comprehensively assess the usability of the Masao Facial Makeup APP, ensuring the reliability and validity of the results. This provides strong data support for further optimization and promotion.

### Design of startup interface and page flow

To align with the user demand for a comfortable, minimalist, and convenient experience, the design process integrates key elements of Masao facial makeup throughout. The key features of the launch interface are clearly presented on the start page, enabling users to efficiently understand the core content of Masao facial makeup. The start page adopts a multi-page layout, primarily using images to capture the user’s attention and spark interest in the APP. Once users enter the start page, the design of the login page is focused on user convenience. Figure 5 presents the detailed login process for the APP. This diagram systematically outlines each step of the login process, user operation options, and information input and verification stages. It helps to further examine the APP’s user access mechanism and security measures, providing a visual reference for subsequent optimization and improvements.

### APP font design and interface layout

The overall font used in the APP is primarily Song type, with numbers and letters rendered in the DIN font. The hierarchy of the text is determined by factors such as size, color, weight, spacing, and special font packages. Given that the APP focuses on displaying a significant amount of text-based information related to ICH, effectively managing text hierarchy is essential to enhancing the user experience. The Chinese text size generally ranges from 12px to 19px, with the main body text set at 14px, subtitles at 16px, text spacing at 10px, and line spacing at 25px. Titles are distinguished from regular text through the use of bold fonts, making it easier for users to differentiate between headings and content. Additionally, text on the jump page is color-coded to facilitate user navigation and improve usability.

The interface layout plays a very important role in APP interface design, and page design principles are used in the design process^12^. Based on the APP frame diagram obtained earlier, the page is designed. Each page contains specific content. In the design process of page layout, the arrangement of page text and sections plays an extremely important role in displaying information in the APP. The page layout of the APP is mainly based on the page layout of sub-modules.

### APP interface conversion design

The dynamic transition effect of an APP significantly influences the user experience, making interface transitions a crucial aspect of APP design^21^. Dynamic transitions are more effective than static ones, as they enhance the overall user experience. In designing dynamic effects, it is important to align with the movement patterns of objects in the real world, mapping users’ perception of object movement into the design of dynamic effects. This creates a dynamic effect that is more intuitive and in sync with user perception. For the design of the Masao Facial Makeup APP, the interactive prototype tool Protopie is primarily used to create unique dynamic effects on the guide page. When transitioning from one interface to another, the page illustration is automatically loaded and gradually enlarged at a consistent speed, providing users with an immersive experience^22–24^.

### Usability test results

**Fig. 10:**
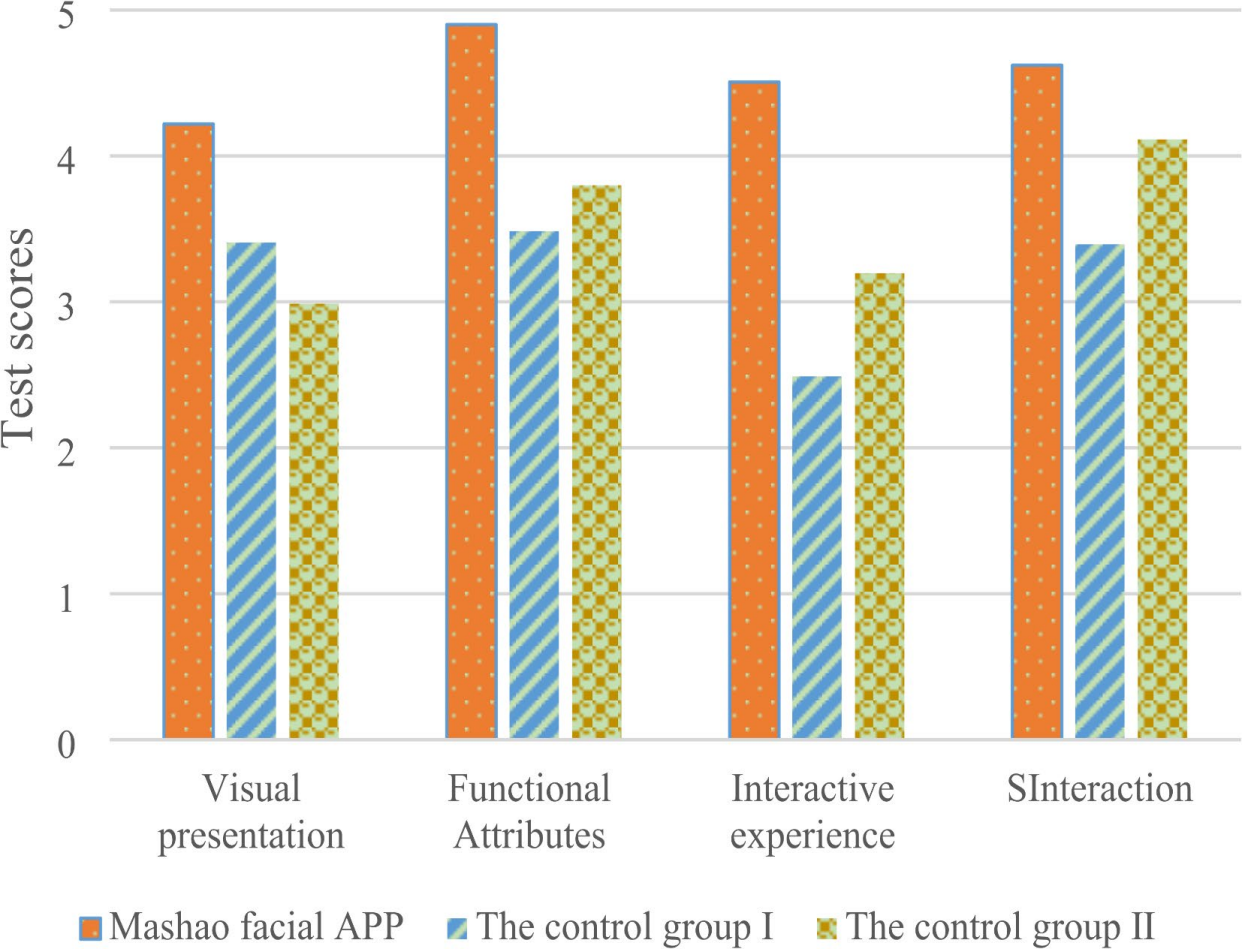
Comparative analysis of evaluation results.

Based on the feasibility score analysis, Fig. 10 presents a comparison of the Masao Facial Makeup APP with Control Group I and Control Group II across various data points. It clearly illustrates the quantitative differences between them in key metrics such as visual presentation, functional attributes, and interactive experience. These data comparisons provide an intuitive basis for an in-depth analysis of the strengths and features of the Masao Facial Makeup APP. It can support further exploration of its potential application value and development prospects in relevant fields, thus establishing an important reference for subsequent research.

Figure 10 shows that the visual effect score of Masao Facial Makeup APP is 4.2, the corresponding score of functional attributes is 4.9, the interactive experience score is 4.5, and the operating experience score is 4.6. Functional attributes show a high score. Compared to the two control groups, the proposed APP demonstrates superior performance, with the average values of the four test indicators significantly higher than those of the other two groups. This indicates that the designed APP interface interaction is highly effective. When compared to Chen’s study^15^, the research here has more precise objectives and a more advanced research direction, offering stronger support for the future development of ICH.

## Discussion

This paper focuses on the design, testing, and analysis of the Masao Facial Makeup APP and has achieved significant results on multiple levels. It makes a positive contribution to the inheritance of Masao facial makeup culture and the development of related fields. In terms of user needs analysis and functional positioning, through a questionnaire survey and in-depth analysis of 300 volunteers, the target user group of the APP is precisely identified. They are individuals aged 15 to 45 who are passionate about traditional culture and cultural inheritance. This group tends to access information through internet channels, aligning with current trends in digital cultural dissemination. Based on this, the identified functional modules (viewing and uploading Masao facial makeup short videos, online courses, community interaction, and product purchasing) comprehensively meet users’ needs in areas like cultural learning, social interaction, and acquiring physical products. Compared to traditional methods of disseminating ICH culture, this user-centric functional design overcomes the limitations of single-channel communication and monotonous content. It provides new ideas and models for the dissemination of ICH culture in the digital age.

In the APP design phase, from interface design to technical implementation, the dual consideration of cultural inheritance and user experience is evident. The choice of the Android system and a minimalist design style, combined with professional software such as Photoshop, ProtoPie, Adobe XD, and Adobe Illustrator, ensures both aesthetic appeal and ease of use. For example, in font design, Song typeface and DIN font are chosen according to the hierarchical structure of the text, with precise control over font size, color, and spacing. This makes the text clear and enhances the user reading experience. The interface layout optimizes the information display based on the APP framework and submodule arrangement principles. In terms of technical implementation, the framework includes a face recognition system, a special effects design system, and a face-special effects matching system. This framework innovatively integrates Masao facial makeup special effects into the APP, achieving a deep fusion of cultural elements and modern technology. This not only enriches the presentation forms of ICH culture but also provides a technological reference for the digital preservation of similar ICH cultures.

Through usability testing with 50 volunteers and comparisons with two groups of traditional culture APPs, the Masao Facial Makeup APP achieves high scores in visual effects (4.2), functionality (4.9), interaction experience (4.5), and operational experience (4.6). It significantly outperforms the control groups. This indicates that the designed APP has clear advantages in terms of functional completeness, ease of operation, visual appeal, and user interaction. From a cultural dissemination perspective, the high functionality score suggests that the APP effectively meets users’ needs for in-depth exploration and learning of the Masao facial makeup culture. For example, through short videos and online courses, users can systematically understand the history, production techniques, and cultural connotations of Masao facial makeup. The positive interaction and operational experiences have facilitated communication and sharing within the community, creating a favorable cultural dissemination environment, and accelerating the spread and depth of the culture within the target audience. The relatively high visual effect score highlights the successful application of Masao facial makeup cultural elements in the interface design. Its unique colors and patterns have captured users’ attention, enhancing the cultural appeal and communicability.

However, this paper also has certain limitations. The design of Masao facial makeup types is not detailed enough and does not fully showcase the diversity and complexity of the Masao facial makeup, which could affect users’ comprehensive and in-depth understanding of the culture. Future research can expand on this aspect by inviting related ICH inheritors and cultural experts to participate. Using advanced technologies such as 3D modeling and virtual reality, high-precision digital models and displays of different types of Masao facial makeup could be created to enrich the APP’s mask resource library. Additionally, interactive explanations could be added, allowing users to closely observe and learn the unique features of each mask. Furthermore, the APP’s functionality could be expanded, such as incorporating personalized recommendation features based on users’ learning behaviors. By analyzing users’ browsing history and preferences, relevant Masao facial makeup cultural content could be accurately pushed to users. Strengthening cooperation with offline cultural institutions and tourist attractions can also be explored, enabling online and offline linkage of cultural activities through the APP. For instance, users could make online reservations to visit Masao facial makeup workshops or participate in offline cultural exhibitions. These enhancements could significantly improve users’ engagement and experience with Masao facial makeup culture, providing more robust support for the inheritance and development of the culture.

## Conclusion

Folk culture is a valuable treasure formed through the accumulation of 5,000 years of Chinese history, holding a pivotal position in the lineage of cultural inheritance. Masao facial makeup, as a unique branch of folk culture, plays an especially critical role in cultural preservation and transmission in the contemporary cultural context. This paper focuses on the development of the Masao Facial Makeup APP, following rigorous theoretical research paradigms and standardized software design processes. During the software interface interaction design process, a systematic survey is conducted using questionnaires to understand user needs. Based on data analysis results, the software’s functions are accurately defined, leading to the design and construction of the software interface. Through feasibility testing, the APP achieves significant results in the dimensions of visual effects, functionality, interaction experience, and operational experience, with scores of 4.2, 4.9, 4.5, and 4.6, respectively. Compared to two existing traditional cultural APPs, it demonstrates clear advantages and shows no abnormalities during the testing period, proving its feasibility and reliability. An in-depth analysis of the test results reveals that, in terms of visual effects, the APP successfully digitalizes the traditional visual elements of Masao facial makeup. Its color system, while preserving the original cultural characteristics, is optimized for adaptation to mobile device screens, presenting a vivid and harmonious visual effect. The intricate patterns, including facial contours and decorative motifs, are clearly displayed, faithfully restoring the artistic essence of Masao facial makeup and providing a valuable reference for the digital visual representation of ICH culture. Regarding functionality, the short video feature fully leverages its communication advantages. Users can watch a variety of Masao facial makeup creation videos to intuitively understand the production techniques and processes. The online course feature provides users with a structured way to learn about the cultural connotations of Masao facial makeup, covering a wide range of knowledge, including historical origins and symbolic meanings. The community interaction feature significantly enhances user engagement and knowledge sharing. Users’ comments and shared insights form a dynamic cultural exchange ecosystem, greatly enriching the depth and breadth of users’ understanding of Masao facial makeup culture. Although this paper has achieved certain results, there is still room for improvement. In terms of collecting and presenting Masao facial makeup types, the current coverage is not extensive enough and does not fully showcase its rich diversity. Future research will strengthen cooperation with ICH inheritors and cultural research institutions to delve deeper into the collection of more Masao facial makeup resources. By employing advanced digital technologies, high-quality presentations of these resources will be created, further enriching the content system of the APP.

### Recommendations

To optimize the Masao Facial Makeup APP, it is recommended to introduce personalized customization features and integrate elements of Baoji folk culture. Developing a web-based version and adapting the APP for smart TVs would also broaden its accessibility. Additionally, establishing a user feedback data analysis system, driven by data, would facilitate ongoing improvements to the APP, enhancing both user experience and cultural dissemination. These measures would contribute to increased user retention and activity, further supporting the inheritance and development of Masao facial makeup culture.

### Contributions

This paper innovatively combines Masao facial makeup with the internet by developing a specialized APP, changing its cultural transmission medium, and expanding its audience. It offers new insights into the digital inheritance of ICH, helps traditional folk culture thrive in modern society, and contributes to the exploration and practice of cultural protection and development.

## Electronic supplementary material

Below is the link to the electronic supplementary material.

Supplementary Material 1

Supplementary Material 2

Supplementary Material 3

## Data Availability

Data is provided within the manuscript or supplementary information files.

## References

[1] Skublewska-Paszkowska, M., Milosz, M., Powroznik, P. & Lukasik, E. 3D technologies for intangible cultural heritage preservation—literature review for selected databases. *Herit. Sci.* **10**(1), 1–24 (2022). 10.1186/s40494-021-00633-x PMC8724653 35003750

[2] Selmanović, E. et al. Improving accessibility to intangible cultural heritage preservation using virtual reality. *J. Comput. Cult. Herit. (JOCCH)* **13**(2), 1–19 (2020).

[3] Xue, K., Li, Y. & Meng, X. An evaluation model to assess the communication effects of intangible cultural heritage. *J. Cult. Herit.* **40**, 124–132 (2019).

[4] Kim, S., Im, D. U., Lee, J. & Choi, H. Utility of digital technologies for the sustainability of intangible cultural heritage (ICH) in Korea. *Sustainability* **11**(21), 6117 (2019).

[5] Su, X., Li, X. & Kang, Y. A bibliometric analysis of research on intangible cultural heritage using CiteSpace. *Sage Open.* **9**(2), 2158244019840119 (2019).

[6] Koutsabasis, P. et al. Co-designing the user experience of location-based games for a network of museums: Involving cultural heritage professionals and local communities. *Multimodal Technol. Interact.* **6**(5), 36 (2022).

[7] Hou, S., Ge, Q. & Liu, Y. Research on digital protection system of intangible cultural heritage based on mobile augmented reality technology. *J. Syst. Simul.* **33**(6), 1334 (2021).

[8] Li, Y. & Duan, P. Research on the innovation of protecting intangible cultural heritage in the internet plus era. *Procedia Comput. Sci.* **154**, 20–25 (2019).

[9] Yan, W. J. & Chiou, S. C. The safeguarding of intangible cultural heritage from the perspective of civic participation: the informal education of Chinese embroidery handicrafts. *Sustainability* **13**(9), 4958 .

[10] Verde, A. & Valero, J. M. Virtual museums and Google arts & culture: Alternatives to the face-to-face visit to experience art. *Int. J. Educ. Res.* **9**(2), 43–54 (2021).

[11] Sarker, I. H., Hoque, M. M., Uddin, M. K. & Alsanoosy, T. Mobile data science and intelligent apps: Concepts, AI-based modeling and research directions. *Mob. Networks Appl.* **26**, 285–303 (2021). 10.1007/s11036-020-01650-z PMC7489576 40477078

[12] Papadakis, S. Tools for evaluating educational apps for young children: A systematic review of the literature. *Interact. Technol. Smart Educ.* **18**(1), 18–49 (2021).

[13] Pettersen, E. F. et al. UCSF ChimeraX: Structure visualization for researchers, educators, and developers. *Protein Sci.* **30**(1), 70–82 (2021). 32881101 10.1002/pro.3943 PMC7737788

[14] Mehra, A., Paul, J. & Kaurav, R. P. S. Determinants of mobile apps adoption among young adults: Theoretical extension and analysis. *J. Mark. Commun.* **27**(5), 481–509 (2021).

[15] Chen, Z. Visualizing experiencescape–from the art of intangible cultural heritage. *Curr. Issues Tourism* **25**(4), 559–578 (2022). .

[16] Ranwa, R. Impact of tourism on intangible cultural heritage: Case of Kalbeliyas from Rajasthan, India. *J. Tourism Cult. Change* **20**(1–2), 20–36 (2022).

[17] Zhang, Y. & Lee, T. J. Alienation and authenticity in intangible cultural heritage tourism production. *Int. J. Tourism Res.* **24**(1), 18–32 (2022).

[18] Meloni, M. et al. Engineering origami: A comprehensive review of recent applications, design methods, and tools. *Adv. Sci.* **8**(13), 2000636 (2021).

[19] Kumar, S. & Shah, A. Revisiting food delivery apps during COVID-19 pandemic? Investigating the role of emotions. *J. Retailing Consumer Serv.* **62**, 102595 (2021).

[20] Yang, Z., Du, H., Jin, L. & Poelman, D. High-performance lead-free bulk ceramics for electrical energy storage applications: Design strategies and challenges. *J. Mater. Chem. A* **9**(34), 18026–18085 (2021).

[21] Pandiangan, S. M. T. Effect of packaging design on repurchase intention to the politeknik IT&B medan using E-Commerce applications. *J. Prod. Oper. Manag. Econom. JPOME)* **2**(01), 15–21, (2022).

[22] Jin, P. & Liu, Y. Fluid space: digitisation of cultural heritage and its media dissemination. *Telematics Inf. Rep.* **8**, 100022 (2022).

[23] Wang, Q. The digitisation of intangible cultural heritage oriented to inheritance and dissemination under the threshold of neural network vision. *Mob .Inf. Syst.* **2022**(1), 6323811 (2022)

[24] Lv, L. Research on the digital dissemination of non-heritage traditional culture in the context of the meta-universe. *Trans. Social Sci. Educ. Humanit. Res.* **2**, 102–107 (2023).

