# Supplementary material for: Using Masao facial makeup in software interface interaction design from the perspective of digital communication
Source: Sci Rep. 2025 Mar 5;15:7680. doi: 10.1038/s41598-025-90448-8 (PMC11882995; doi:10.1038/s41598-025-90448-8)
Supplement: Supplementary file 2 — Supplementary Material 2 [file 41598_2025_90448_MOESM2_ESM.docx]

**Code：**

import pandas as pd

import numpy as np

from scipy.stats import ttest_ind

# Assume there is a CSV file storing the questionnaire data. The following is an example code for reading the data.

# In actual use, modify it according to the real format and storage path of the questionnaire data.

data = pd.read_csv('masao_facial_makeup_questionnaire_data.csv')

# Extract relevant column data. Assume the column names correspond to the functional requirements and evaluation dimensions in the paper.

# For example, 'watch_video' represents the evaluation of the short video watching function, 'visual_effect' represents the visual effect evaluation, etc.

# Adjust the actual column names according to the data situation.

watch_video = data['watch_video']

visual_effect = data['visual_effect']

functional_attr = data['functional_attr']

interactive_exp = data['interactive_exp']

operation_exp = data['operation_exp']

# Calculate the mean, median, and standard deviation of each dimension

mean_watch_video = np.mean(watch_video)

median_watch_video = np.median(watch_video)

std_watch_video = np.std(watch_video)

mean_visual_effect = np.mean(visual_effect)

median_visual_effect = np.median(visual_effect)

std_visual_effect = np.std(visual_effect)

mean_functional_attr = np.mean(functional_attr)

median_functional_attr = np.median(functional_attr)

std_functional_attr = np.std(functional_attr)

mean_interactive_exp = np.mean(interactive_exp)

median_interactive_exp = np.median(interactive_exp)

std_interactive_exp = np.std(interactive_exp)

mean_operation_exp = np.mean(operation_exp)

median_operation_exp = np.median(operation_exp)

std_operation_exp = np.std(operation_exp)

# Correlation analysis. Take the visual effect and interactive experience as an example to calculate the Pearson correlation coefficient.

# In the actual analysis, it can be extended to the correlation analysis between more dimensions as needed.

correlation_matrix = np.corrcoef(visual_effect, interactive_exp)

correlation_coefficient = correlation_matrix[0, 1]

# Hypothesis testing. Compare the score differences between this APP and the two control groups in each dimension.

# Take the visual effect as an example to perform an independent sample t-test between the APP and Control Group I and Control Group II.

# Similar tests need to be performed for each evaluation dimension in actual use.

app_visual = visual_effect[data['app_type'] == 'Masao Facial Makeup APP']

control1_visual = visual_effect[data['app_type'] == 'Control Group I']

control2_visual = visual_effect[data['app_type'] == 'Control Group II']

t_statistic_visual, p_value_visual = ttest_ind(app_visual, control1_visual)

t_statistic_visual_2, p_value_visual_2 = ttest_ind(app_visual, control2_visual)

# Output the results

print("Short video function:")

print("Mean:", mean_watch_video)

print("Median:", median_watch_video)

print("Standard deviation:", std_watch_video)

print("Visual effect:")

print("Mean:", mean_visual_effect)

print("Median:", median_visual_effect)

print("Standard deviation:", std_visual_effect)

print("Correlation coefficient with interactive experience:", correlation_coefficient)

print("t-test statistic with Control Group I:", t_statistic_visual)

print("p-value with Control Group I:", p_value_visual)

print("t-test statistic with Control Group II:", t_statistic_visual_2)

print("p-value with Control Group II:", p_value_visual_2)

print("Functional attributes:")

print("Mean:", mean_functional_attr)

print("Median:", median_functional_attr)

print("Standard deviation:", std_functional_attr)

print("Interactive experience:")

print("Mean:", mean_interactive_exp)

print("Median:", median_interactive_exp)

print("Standard deviation:", std_interactive_exp)

print("Operation experience:")

print("Mean:", mean_operation_exp)

print("Median:", median_operation_exp)

print("Standard deviation:", std_operation_exp)

**Description：**

This document provides a detailed explanation of the computational code designed for the evaluation of the Masao Facial Makeup APP. The code is developed based on the research requirements and aims to analyze the data collected from user questionnaires to assess the performance of the APP in various aspects.

Data Reading and Preparation

The code begins with importing necessary libraries such as `pandas` for data manipulation and `numpy` for numerical computations. The data is assumed to be stored in a CSV file named'masao_facial_makeup_questionnaire_data.csv'. Using the `read_csv` function from `pandas`, the data is loaded into a `DataFrame` object named `data`. Subsequently, relevant columns corresponding to different evaluation dimensions like 'watch_video' for the short video function, 'visual_effect' for the visual appearance, 'functional_attr' for the functionality, 'interactive_exp' for the interaction experience, and 'operation_exp' for the operational experience are extracted from the `DataFrame`.

Descriptive Statistics Calculation

For each of the extracted columns, the mean, median, and standard deviation are calculated. The `mean` function from `numpy` is used to compute the average value of the data in each column, providing an overall measure of the central tendency. The `median` function calculates the middle value of the sorted data, which helps to understand the distribution and is less affected by extreme values. The `std` function calculates the standard deviation, indicating the dispersion of the data points around the mean. These descriptive statistics offer a comprehensive understanding of the users' responses in each evaluation dimension.

Correlation Analysis

To explore the relationships between different dimensions, a correlation analysis is conducted. Taking the visual effect and interactive experience as an example, the `corrcoef` function from `numpy` is employed to calculate the Pearson correlation coefficient. This coefficient ranges from -1 to 1, where a value close to 1 indicates a strong positive linear relationship, meaning that an improvement in the visual effect is likely to be associated with a better interactive experience. A value close to -1 implies a negative linear relationship, and a value close to 0 suggests little or no linear correlation.

Hypothesis Testing

Hypothesis testing is a crucial part of the evaluation to compare the performance of the Masao Facial Makeup APP with that of the control groups. In the code, independent sample t-tests are performed for each evaluation dimension. For instance, in the case of the visual effect dimension, the data of the APP and the two control groups (Control Group I and Control Group II) are separated based on the 'app_type' column in the data. Then, the `ttest_ind` function from `scipy.stats` is used to conduct the t-tests. The t-test statistic and the corresponding p-value are calculated. A small p-value (usually less than 0.05) indicates that there is a significant difference between the APP and the control group, suggesting that the APP performs better or worse in that particular dimension depending on the sign of the t-test statistic.

In conclusion, this computational code provides a comprehensive framework for analyzing the data related to the Masao Facial Makeup APP. By calculating descriptive statistics, conducting correlation analysis, and performing hypothesis testing, it helps to evaluate the APP's performance objectively and provides valuable insights for further improvement and research. However, it should be noted that the code is based on the assumed data format and structure, and adjustments may be required according to the actual data situation in real applications.
