# Supplementary material for: Using Masao facial makeup in software interface interaction design from the perspective of digital communication
Source: Sci Rep. 2025 Mar 5;15:7680. doi: 10.1038/s41598-025-90448-8 (PMC11882995; doi:10.1038/s41598-025-90448-8)
Supplement: Supplementary file 3 — Supplementary Material 3 [file 41598_2025_90448_MOESM3_ESM.docx]

**Appendix**

User Demand Survey for Masao Facial Makeup APP

Dear Sir/Madam,

Thank you for taking the time to participate in this survey regarding the Masao Facial Makeup APP. The purpose of this survey is to understand your knowledge, interest, and expectations for the features of the APP, so that we can better develop and optimize the Masao Facial Makeup APP and promote the transmission and inheritance of this intangible cultural heritage (ICH). Your responses will provide crucial insights for our research, and all information will be kept confidential and used solely for academic purposes.

**I. Personal Information**

1. What is your gender?

A. Male

B. Female

2. What is your age?

A. 15-20 years

B. 21-35 years

C. 36-45 years

D. 46 years or older

3. What is your occupation?

A. Government or corporate employee

B. Private enterprise employee

C. Foreign enterprise employee

D. Other (please specify)

4. What mobile operating system do you use?

A. iOS

B. Android

C. Other (please specify)

**II. Knowledge and Exposure to Masao Facial Makeup**

1. Are you familiar with the Masao facial makeup?

A. Not at all

B. A little

C. Familiar

D. Very familiar

2. If you are familiar with Masao facial makeup, how did you first come across it? (Multiple choices allowed)

A. Books or newspapers

B. Television

C. Internet

D. School learning

E. Travel

F. Other (please specify)

3. What do you think is the biggest challenge in protecting Masao facial makeup?

A. Lack of a comprehensive protection mechanism

B. Insufficient dissemination

C. Lack of inheritors

D. Lack of funding

**III. APP Usage Willingness and Expectations**

1. Would you be willing to download the Masao Facial Makeup APP on your mobile device?

A. Yes

B. No

2. Which aspects of Masao facial makeup do you value the most? (Multiple choices allowed)

A. Fun

B. Educational significance

C. Artistic value

D. Cultural connotation

3. What do you think is the most important feature of the Masao Facial Makeup APP? (Multiple choices allowed)

A. Rich content (such as introducing different types of Masao facial makeup and historical stories)

B. Simple and smooth operation (such as one-click video watching, and quick switching between pages)

C. Comprehensive functionality (including, but not limited to, video learning, interactive communication, and product purchasing)

D. High interactivity (enabling communication with other users and ICH inheritors)

E. Aesthetic interface (coordinated color scheme, clear display of patterns, and distinct Masao facial makeup features)

F. Other (please specify)

4. If there were an APP for the protection of Masao facial makeup, what features would you want it to have? (Multiple choices allowed)

A. Short videos (such as detailed demonstrations of the Masao facial makeup production process, from material preparation to painting techniques and finishing, and videos of Masao facial makeup performances)

B. Handcraft tutorials (detailed drawing steps by professional ICH inheritors, technique demonstrations, with accompanying text instructions and image examples)

C. Offline experience course registration and information inquiries (providing course schedules, locations, content descriptions, and registration channels)

D. Community communication (users can share their Masao facial makeup creation experiences, exhibition visits, and understandings of the culture; ask questions and receive answers within the community; interact with ICH inheritors)

E. ICH product sales (selling Masao Facial Makeup-related craft products and lifestyle items featuring Masao facial makeup, such as tea sets, ceramics, mirrors, and pendants, with product descriptions and user reviews)

F. Masao facial makeup knowledge quizzes and tests (regular knowledge quizzes on the history, culture, and creation of Masao facial makeup, with results and explanations)

G. Location-based recommendations and navigation for Masao facial makeup cultural sites (recommend nearby cultural centers, workshops, and exhibitions related to Masao facial makeup, with navigation features)

H. Masao facial makeup cultural events information and registration (information on Masao Facial Makeup exhibitions and cultural festivals, with online registration)

I. Personalized recommendations (precise content delivery based on user's browsing history, saved items, and interactions)

**IV. Traditional Culture APP User Experience**

1. Have you ever used other traditional culture-related APPs?

A. Yes

B. No

2. If you have used such APPs, what do you think are the shortcomings of existing traditional culture APPs? (Multiple choices allowed)

A. Insufficient content (such as shallow cultural knowledge and single presentation format)

B. Unattractive interface (such as uncoordinated colors, layouts, and lack of cultural elements)

C. Lack of appeal (unable to sustain user interest)

D. False advertising (actual functionality differs from promotional claims)

E. Poor interactivity (poor communication channels between users and users and between users and developers)

F. Outdated features (failure to support new technologies, slow updates)

G. Insufficient cultural depth (lack of thorough explanation of cultural meaning and historical significance)

H. Slow loading speed

I. Too many ads

Thank you again for your support and cooperation!
